# BMI Percentile Cutoffs for Overweight and Obesity Are Set Too High in Terms of Adiposity and Metabolic Markers for Asian Children and Adolescents

**DOI:** 10.1111/ijpo.70075

**Published:** 2025-12-10

**Authors:** Suk‐Jin Hong, Xinlian Zhang, Phillipp Hartmann

**Affiliations:** ^1^ Department of Pediatrics University of California La Jolla California USA; ^2^ Department of Pediatrics, School of Medicine Daegu Catholic University Daegu Korea; ^3^ Division of Biostatistics and Bioinformatics, Herbert Wertheim School of Public Health and Human Longevity Science University of California San Diego San Diego California USA; ^4^ Division of Gastroenterology, Hepatology & Nutrition, Rady Children's Hospital San Diego San Diego California USA

**Keywords:** body mass index, ethnicities, NHANES

## Abstract

**Objectives:**

Asian adults develop metabolic complications at lower body mass indices (BMIs) compared with other ethnic groups, leading to lower BMI cutoffs for overweight and obesity. However, it remains unclear whether such adjustments are warranted in Asian children and adolescents. We aimed to determine whether lower BMI percentile cutoffs should be considered for overweight and obesity among Asian children and adolescents in the United States.

**Methods:**

We analysed nationally representative data from U.S. children and adolescents from NHANES, 2012–2020. Metabolic parameters—including truncal fat percentage, glycemic markers, lipid profiles and liver disease markers—were compared between Asian and non‐Asian participants within the 85–95th percentile and ≥ 95th percentile ranges. Metabolic parameter‐based matching analyses identified corresponding BMI percentiles between groups.

**Results:**

Asian children had more pronounced metabolic abnormalities than non‐Asians in the overweight and obesity groups. After matching for metabolic parameters, Asians had the same levels of metabolic dysfunction at lower BMI percentiles than non‐Asians, with averaged differences of approximately 3–9 BMI percentiles across various BMI percentile ranges.

**Conclusions:**

Asian children and adolescents experience elevated metabolic risks at currently defined BMI percentiles. Lower BMI cutoffs targeting the 80th percentile for overweight and 90th percentile for obesity may improve early detection of metabolic risks in the Asian paediatric population.

**Abbreviations**
BMI
: body mass index
CAP: controlled attenuation parameter
CDC: centers for disease control and prevention
DXA: dual‐energy X‐ray absorptiometry
HDL: high‐density lipoprotein
HOMA‐IR: homeostatic model assessment of insulin resistance
IQR: interquartile range
LDL: low‐density lipoprotein
MASLD: metabolic dysfunction‐associated steatotic liver disease
NHANES: national health and nutrition examination survey
T2DM: type 2 diabetes mellitus

## 1 Introduction

According to the United States (U.S.) 2020 Census, approximately 4.4 million individuals under the age of 18 were identified as Asian [1]. The National Health and Nutrition Examination Survey (NHANES) data indicated that the prevalence of obesity among Asian children and adolescents ranged from 8.6% to 11.0% between 2011 and 2018 [2]. Childhood obesity is associated with adverse health outcomes, including hypertension, dyslipidaemia, type 2 diabetes mellitus (T2DM) and metabolic dysfunction‐associated steatotic liver disease (MASLD) [3, 4, 5, 6]. Therefore, accurate screening and early identification are crucial, making appropriate body mass index (BMI) percentile cutoffs essential for effective prevention strategies. Despite its limitations, the BMI remains the most widely used and practical screening tool for identifying children and adolescents at risk for obesity and its complications [7].

Internationally, including by the World Health Organisation (WHO) and the U.S. Centers for Disease Control and Prevention (CDC), overweight in adults is defined as a BMI of 25–29.9 kg/m^2^ and obesity as BMI ≥ 30 kg/m^2^ [8, 9]. However, BMI‐related risks vary by race and ethnicity [10, 11]. A WHO expert consultation noted that Asian adults face increased risks of T2DM and cardiovascular diseases at lower BMI thresholds [12]. Additionally, the Western Pacific Regional Office of WHO (WPRO) proposed lower cutoffs for Asians: BMI ≥ 23.0 kg/m^2^ for overweight and BMI ≥ 25.0 kg/m^2^ for obesity [13]. Similarly, obesity expert groups from South and Southeast Asia have provided consensus recommendations, defining overweight as BMI ≥ 23.0 kg/m^2^ and obesity as BMI ≥ 25.0 kg/m^2^ for South and Southeast Asian populations [14]. Such adjustments are based on evidence that Asian populations have higher central fat percentages at lower BMI values compared with non‐Asian populations, contributing to greater obesity‐related health risks at lower BMI thresholds [12, 13, 14].

Studies have shown that BMI underestimates body fat, particularly visceral adiposity, in Asian children compared with their White counterparts, hampering the detection of metabolic syndrome and MASLD [10, 15, 16]. This highlights the need for race‐ and ethnicity‐specific paediatric BMI thresholds, similar to those established for adults. For the paediatric population, overweight and obesity are generally defined as 85th to < 95th and ≥ 95th BMI percentiles for age and sex. The U.S. CDC employs the same BMI percentile cutoffs; however, these are based on general population references that do not account for ethnic variations in body composition and fat distribution. Given the potential for underdiagnosis of obesity‐related health risks in Asian children and adolescents, there is a critical need to examine whether existing BMI percentile cutoffs are appropriate for this population. Unlike in adults, BMI percentile adjustments for Asian children and adolescents have not been clearly established.

This study aims to compare BMI percentiles associated with metabolic health among Asian children and adolescents versus children and adolescents of other races and ethnicities, and to determine whether lower BMI percentile thresholds should be applied to Asian children and adolescents for more accurate screening for obesity‐related metabolic risks and complications.

## 2 Materials and Methods

### 2.1 Data Source and Study Population

The NHANES is a nationally representative, cross‐sectional survey conducted on U.S. residents every 2 years. Managed by the National Center for Health Statistics and CDC, NHANES aims to assess the health and nutritional status of the U.S. population. As part of the survey, participants provide demographic information, undergo a physical examination, blood and imaging studies and complete validated questionnaires about their health behaviours [17].

For this study, we obtained the NHANES datasets 2011–2012 (referred to as the ‘NHANES 2012’ dataset), 2013–2014 (‘NHANES 2014’), 2015–2016 (‘NHANES 2016’), 2017–2018 (‘NHANES 2018’) and 2017–2020 (‘NHANES 2020’) using the ‘nhanesA’ library in R [18]. NHANES is cross‐sectional and there is no overlap in participants across the study periods. The study population included 17 096 participants aged 2–18 years who had data on BMI and race and ethnicity (including non‐Hispanic Asians), and—as available—metabolic parameters of interest. The number of participants per dataset was: NHANES 2012 (*n* = 3213), NHANES 2014 (*n* = 3394), NHANES 2016 (*n* = 3225), NHANES 2018 (*n* = 2704) and NHANES 2020 (*n* = 4560). Sample sizes varied by measurement because NHANES employs a complex sampling design, with certain variables measured only within specific age groups or selected subsamples (e.g., morning fasting subsample) [19].

### 2.2 Metabolic Parameters

From the NHANES dataset [17], we selected metabolic parameters associated with metabolic dysfunction and obesity‐related complications, categorising them into four groups: truncal fat percentage measured by dual‐energy X‐ray absorptiometry (DXA or DEXA); glucose metabolism and insulin resistance, including haemoglobin A1c (HbA1c), fasting glucose, fasting insulin and homeostatic model assessment for insulin resistance (HOMA‐IR) as the product of fasting insulin [μU/mL] * fasting glucose [mg/dL]/405; lipid profiles, including total cholesterol, high‐density lipoprotein cholesterol (HDL‐C), fasting triglycerides (TG) and fasting low‐density lipoprotein cholesterol (LDL‐C); and liver fat and fibrosis assessed using controlled attenuation parameter (CAP) values and liver stiffness measurements via transient elastography (Fibroscan), respectively [20, 21, 22, 23, 24].

### 2.3 Race and Ethnicity Classification and BMI Percentiles

Race and ethnicity were reported by adult family members and categorised according to NHANES classifications as non‐Hispanic Asian, non‐Hispanic White, non‐Hispanic Black, Mexican American, other Hispanic and other race—including multi‐racial individuals. According to the NHANES Analytic Guidelines, the non‐Hispanic Asian category comprises individuals originating from the Far East, Southeast Asia, or the Indian subcontinent (e.g., Cambodia, China, India, Japan, Korea, Malaysia, Pakistan, Philippines, Thailand and Vietnam) but does not provide further differentiation within the Asian subgroup [19]. BMI was calculated as weight in kilograms divided by height in meters squared (kg/m^2^). Updated age‐ and sex‐specific BMI percentiles were calculated using the ‘cdcanthro’ library in R.

### 2.4 Cohort Matching

We employed a two‐step matching approach to enable comparisons between Asian and non‐Asian groups. First, we performed a simple comparison of BMI and metabolic parameters across racial and ethnic groups without matching, using Asians as the reference category. Second, to rigorously evaluate metabolic differences independent of BMI distribution, we performed propensity score matching using the MatchIt package in R [25]. Propensity score matching was conducted using the nearest neighbour method without replacement, with a matching ratio of 1:1 and a calliper width set to 0.1. BMI percentile was used as the primary matching variable. The performance of propensity score matching was evaluated by comparing median differences and interquartile ranges (IQR) of the matching variable between groups after matching, confirming adequate balance.

Specifically, we conducted two distinct matching analyses:

1. BMI percentile matching:

For comparisons between Asian and non‐Asian groups within traditional overweight (85–95th percentile) and obesity (95–99th percentile) categories, we matched individuals by BMI percentiles. This ensured observed differences in metabolic parameters were independent of BMI distribution differences between groups.

2 Metabolic parameter‐based matching:

To identify corresponding BMI percentiles associated with similar metabolic dysfunction between Asian and non‐Asian groups, we conducted matching analyses within three specific BMI percentile ranges (75–85th, 80–90th and 85–95th). Although the 85–95th percentile corresponds to conventional overweight criteria, we additionally included narrower and slightly lower BMI percentile ranges (75–85th, 80–90th) to more precisely identify at which lower BMI percentiles Asian participants began to exhibit metabolic dysfunction comparable to non‐Asians. Asian participants were matched 1:1 to non‐Asian participants based on comparable metabolic profiles. To ensure sufficient matching and comparability, non‐Asian participants were allowed to have BMI percentiles up to approximately 10–15 percentiles lower than the lower boundary of each specified Asian BMI percentile range.

In addition, to address potential confounding by age and sex, we performed supplementary matching analyses, in which BMI percentile, age and sex were simultaneously included as matching variables.

### 2.5 Diagnostic Performance of BMI Percentile Cutoffs for Detecting Metabolic Dysfunction

Diagnostic performance of BMI percentile cutoffs (80, 85, 90 and 95th percentiles) for identifying prediabetes/diabetes, dyslipidaemia and hepatic steatosis among Asian participants was evaluated using sensitivity, specificity, Youden index and area under the receiver operating characteristic curve (AUC, 95% CI) for each cutoff. Presence of prediabetes and diabetes was defined as HbA1c ≥ 5.7%; dyslipidaemia was defined as total cholesterol ≥ 170 mg/dL or HDL < 40 mg/dL; and hepatic steatosis as a controlled attenuation parameter (CAP) ≥ 277 dB/m [26].

### 2.6 Statistical Analysis

Continuous variables were expressed as medians with IQR due to non‐normal distributions. Comparisons between two groups for continuous variables were made using the Wilcoxon‐Whitney–Mann rank‐sum test unless specified differently. For comparisons among three or more groups, the Kruskal‐Wallis test was used. If the Kruskal‐Wallis test indicated statistical significance, pairwise Wilcoxon‐Whitney–Mann rank‐sum tests were conducted, and *p* values were adjusted using the Holm method to control for multiple comparisons and potential false positives. For metabolic parameter group–level summaries, Hodges–Lehmann (HL) estimates, 95% confidence intervals, and *p* values from individual metabolic markers were first obtained using the Wilcoxon rank‐sum test and then averaged across markers within each group, whereas *p* values were combined using Fisher's method to derive a single group‐level *p* value. Metabolic group–specific averages (Truncal Fat, Glucose/Insulin, Lipids and Liver) were computed by equally weighting each group, as inclusion of all metabolic markers in a single matching model would have overrepresented the Glucose/Insulin and Lipid markers. Sample sizes for each group represent the sum of the individual markers within that group.

Significant adjusted *p* values were reported in the tables and figures. All statistical tests were two‐sided and a *p* value of less than 0.05 was considered statistically significant. Statistical analyses were performed using R statistical software, version 4.4.1 (R Foundation for Statistical Computing). ChatGPT has been used solely for language editing to improve the readability of the manuscript.

## 3 Results

### 3.1 Demographics and Clinical Data of the Study Cohort

Table 1 summarises a wide range of demographic, physical examination, laboratory, and imaging data obtained from the NHANES dataset. The cohort encompassed 17 096 individuals. The sex distribution was almost evenly split between male (50.7%) and female (49.3%). The median age among the cohort was 9.5 years. Regarding race and ethnicity, the study population included Non‐Hispanic Asian (9.9%), non‐Hispanic Whites (27.7%), non‐Hispanic Blacks (25.8%), Mexican Americans (18.7%), other Hispanics (10.2%), and children from other racial backgrounds, including multi‐racial individuals (7.7%). The median BMI was recorded at 18.3 kg/m^2^ (interquartile range [IQR], 16.1–22.6 kg/m^2^), whereas the median waist circumference measured 65.1 cm (IQR 54.3–77.7 cm). Key metabolic parameters included HbA1c (median 5.3%, IQR 5.10%–5.5%), fasting glucose (median 96.0 mg/dL, IQR 91.0–100 mg/dL), fasting insulin (median 11.1 μU/mL, IQR 7.37–17.2 μU/mL) and HOMA‐IR (median 2.61, IQR 1.71–4.09). Lipid profiles showed a median total cholesterol of 154 mg/dL (IQR 137–173 mg/dL), fasting triglyceride level of 61.0 mg/dL (IQR 44.0–86.0 mg/dL), fasting LDL‐cholesterol of 85.0 mg/dL (IQR 69.0–102 mg/dL) and HDL‐cholesterol of 53.0 mg/dL (IQR 45.0–61.0 mg/dL). Lastly, Fibroscan results indicated a median of the liver fat marker CAP of 216 dB/m (IQR 186–253 dB/m) and of the indirect liver fibrosis marker liver stiffness of 4.70 kPa (IQR 4.0–5.7 kPa) (Table 1).

**TABLE 1:** Overview of demographic, physical exam, laboratory, and imaging findings in the study population from NHANES 2012 through 2020.

	*n*	Total, *n* = 17 096
Sex	17 096	
Male		8662 (50.7%)
Female		8434 (49.3%)
Age [years]	17 096	9.50 [5.50; 14.5]
Race and Ethnicity	17 096	
Non‐Hispanic Asian		1685 (9.9%)
Non‐Hispanic White		4730 (27.7%)
Non‐Hispanic Black		4415 (25.8%)
Mexican American		3199 (18.7%)
Other Hispanic		1741 (10.2%)
Other Race—including multi‐Racial		1326 (7.7%)
Height [cm]	17 096	139 [115; 160]
Body weight [kg]	17 096	35.7 [21.2; 57.0]
Body mass index percentile	17 096	72 [41.6; 92.2]
Waist circumference [cm]	16 226	65.1 [54.3; 77.7]
Truncal fat [%]	6761	24.9 [18.4; 33.1]
Alanine aminotransferase [IU/L]	5248	15.0 [12.0; 20.0]
Aspartate aminotransferase [IU/L]	5242	21.0 [17.0; 24.0]
Haemoglobin A1c [%]	5421	5.30 [5.10; 5.50]
Fasting glucose [mg/dL]	2521	96.0 [91.0; 100]
Fasting insulin [μU/mL]	2413	11.1 [7.37; 17.2]
HOMA‐IR	2412	2.61 [1.71; 4.09]
Total cholesterol [mg/dL]	10 388	154 [137; 173]
Fasting triglycerides [mg/dL]	2428	61.0 [44.0; 86.0]
Fasting LDL‐cholesterol [mg/dL]	2423	85.0 [69.0; 102]
HDL‐cholesterol [mg/dL]	10 389	53.0 [45.0; 61.0]
Controlled attenuation parameter (CAP) [dB/m]	2520	216 [186; 253]
Liver stiffness [kPa]	2522	4.70 [4.00; 5.70]

*Note:* Values are presented as median and in brackets the first and third quartiles.

Abbreviations: HDL, high‐density lipoprotein; HOMA‐IR, homeostatic model assessment of insulin resistance; LDL, low‐density lipoprotein.

Age and sex distributions by race and ethnicity are summarised in Table S1. Median ages were comparable across racial and ethnic groups, ranging from 9.5 to 10.5 years and the proportions of male and female participants were approximately balanced in all groups.

### 3.2 Overall Metabolic Differences Across All BMI Percentiles

Examination of BMI percentiles across races and ethnicities revealed that Asians had significantly lower BMI percentiles compared with all other racial and ethnic groups, with a notably large difference (medians: Asian 52.62th vs. other races and ethnicities 69.40–80.53th; all *p* < 0.001; Figure S1A). Detailed results regarding metabolic markers are summarized in Figure S1. Despite a markedly lower BMI percentile, Asians still showed significantly higher truncal fat versus non‐Hispanic Blacks (Figure S1B), a higher HbA1c level versus non‐Hispanic Whites (Figure S1C), and a higher total cholesterol level than all other races and ethnicities (Figure S1D). Propensity score matching allows comparison of variables between two groups after matching based on other variables, thus allowing for a more balanced comparison by accounting for potential confounding variables [25]. When metabolic factors between Asian and non‐Asian children and adolescents across all BMI percentiles after matching for BMI percentiles were compared (Figure S2), Asians demonstrated higher truncal fat, HbA1c, fasting triglycerides, total cholesterol and fasting LDL‐cholesterol compared with non‐Asians (Figure S2B–D). When additional matching simultaneously including BMI percentile, age and sex was performed (Figure S3), overall trends remained consistent with the primary analysis. Only two parameters showed different statistical outcomes: LDL‐cholesterol levels were still higher in Asians but no longer significant (*p* = 0.13 vs. *p* = 0.044 previously), whereas CAP values, previously nonsignificant (*p* = 0.12), became significantly higher in Asians (*p* = 0.047).

### 3.3 Metabolic Differences Between Asians and Other Races and Ethnicities in Traditional Overweight Range

**FIGURE 1:**
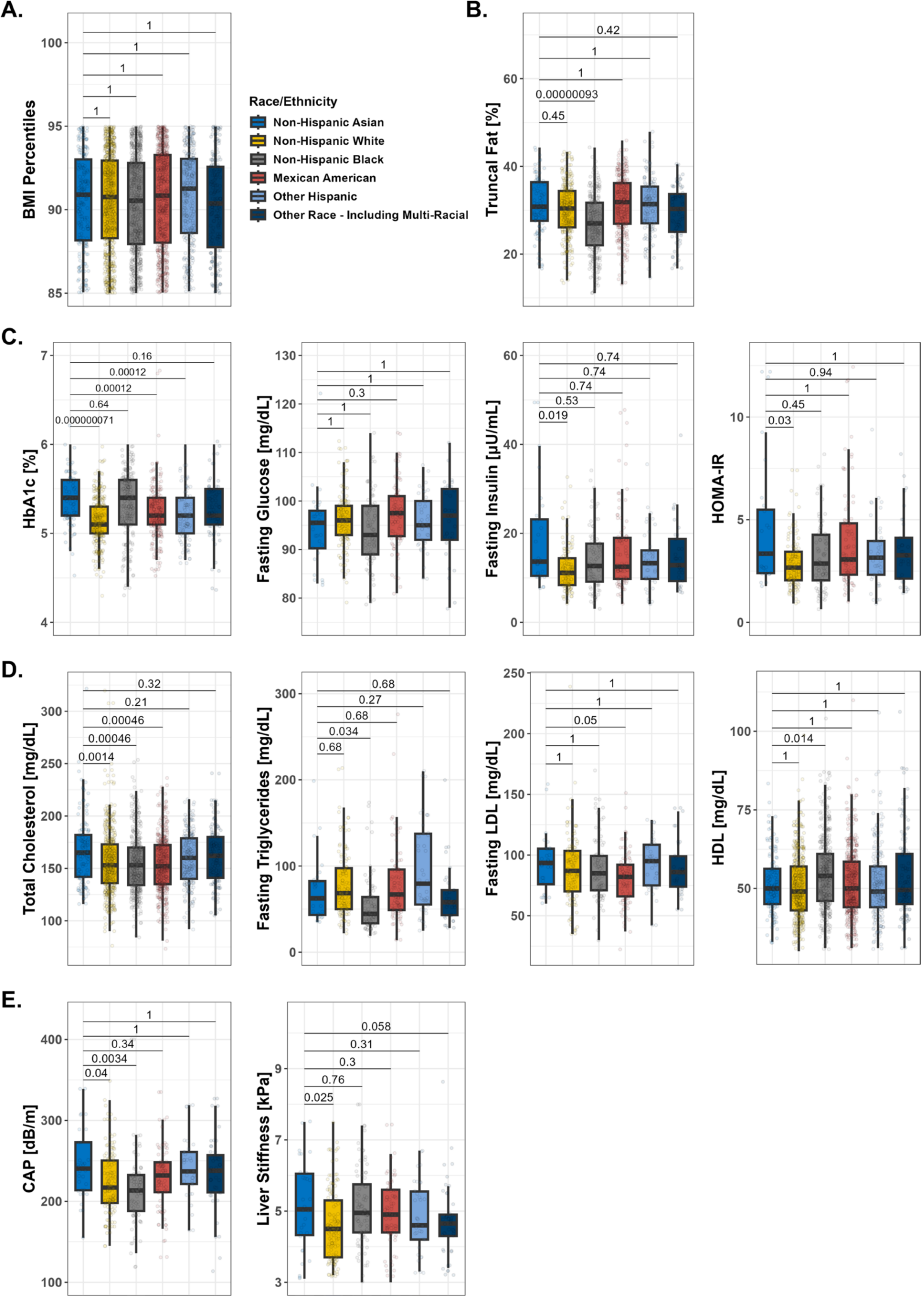
Asian children and adolescents demonstrate more adverse metabolic markers than other races and ethnicities in the 85–95th BMI percentile range. Comparison of metabolic factors by ethnic groups in children and adolescents in the 85–95th BMI percentile range. (A) BMI percentiles (*n* = 2760). (B) Truncal fat percentage (*n* = 1162). (C) Glucose metabolism and insulin resistance: HbA1c (*n* = 949), values in 4%–7% range depicted for better visualisation; fasting glucose (*n* = 421), values in 75–130 mg/dL range depicted; fasting insulin (*n* = 404), values in 0–60 μU/mL range depicted; HOMA‐IR (*n* = 403), values in 0–13 range depicted. (D) Lipid profiles: Total cholesterol (*n* = 1781); fasting triglycerides (*n* = 404); fasting LDL‐cholesterol (*n* = 404); HDL‐cholesterol (*n* = 1781). (E) Liver fat and fibrosis: CAP (*n* = 427); liver stiffness (*n* = 427), values in 3–10 kPa range depicted. *p* values were adjusted for multiple comparisons using the Holm method, with non‐Hispanic Asians serving as the reference group. Boxplots show median, IQR, and whiskers extending to the largest and smallest values within 1.5 × IQR. Abbreviation: BMI, body mass index; CAP, controlled attenuation parameter; HDL, high‐density lipoprotein; HOMA‐IR, homeostatic model assessment of insulin resistance; IQR, indicates interquartile range; LDL, low‐density lipoprotein.

We next analyzed metabolic factors between Asians and other races and ethnicities within the 85–95th BMI percentile range (Figure 1). The BMI percentiles were similar across all ethnic groups (Mexican American: 90.85th, other Hispanic: 91.26th, non‐Hispanic White: 90.76th, non‐Hispanic Black: 90.54th, non‐Hispanic Asian: 90.90th, other Race: 90.37th; all adjusted *p* values = 1.0; Figure 1A). Asians had significantly higher truncal fat percentages compared with non‐Hispanic Blacks (median: 30.8% vs. 27.0%) (Figure 1B). Further, Asians exhibited significantly higher HbA1c levels compared with Mexican Americans, other Hispanics and non‐Hispanic Whites (median: 5.4% vs. 5.2% vs. 5.2% vs. 5.1%, respectively), whereas fasting glucose levels did not differ significantly between Asians and any other groups (Figure 1C). Asians tended to have higher fasting insulin levels and HOMA‐IR values compared with all other groups, with a significant difference observed only when compared with non‐Hispanic Whites (median fasting insulin: 13.6 μU/mL vs. 11.1 μU/mL; median HOMA‐IR: 3.35 vs. 2.67; Figure 1D). With regard to lipid profiles, Asians had significantly higher fasting triglyceride levels compared with non‐Hispanic Blacks (median: 62.5 mg/dL vs. 44.5 mg/dL) (Figure 1D). Asians showed generally higher total cholesterol levels compared with all other groups, with significant differences observed when compared with Mexican Americans, non‐Hispanic Whites and non‐Hispanic Blacks (median: 165.0 mg/dL vs. 152.0 mg/dL vs. 153.0 mg/dL vs. 153.0 mg/dL, respectively) (Figure 1D). There were no significant differences in fasting LDL‐cholesterol levels between Asians and all other groups, although levels were borderline higher compared with Mexican Americans (median: 93.5 mg/dL vs. 82.0 mg/dL; *p* = 0.05; Figure 1D). Non‐Hispanic Blacks had significantly higher HDL‐cholesterol levels compared with Asians (median: 54.0 mg/dL vs. 50.0 mg/dL) (Figure 1D). Regarding liver health, Asians had significantly higher CAP values than non‐Hispanic Whites and non‐Hispanic Blacks (median: 240.5 dB/m vs. 217.0 dB/m vs. 213.5 dB/m, respectively) and significantly higher liver stiffness measurements than non‐Hispanic Whites and multi‐racial individuals (median: 5.2 kPa vs. 4.45 kPa vs. 4.55 kPa, respectively) (Figure 1E).

### 3.4 Metabolic Differences Between Asians and All Non‐Asian Races and Ethnicities Combined in Traditional Overweight Range

**FIGURE 2:**
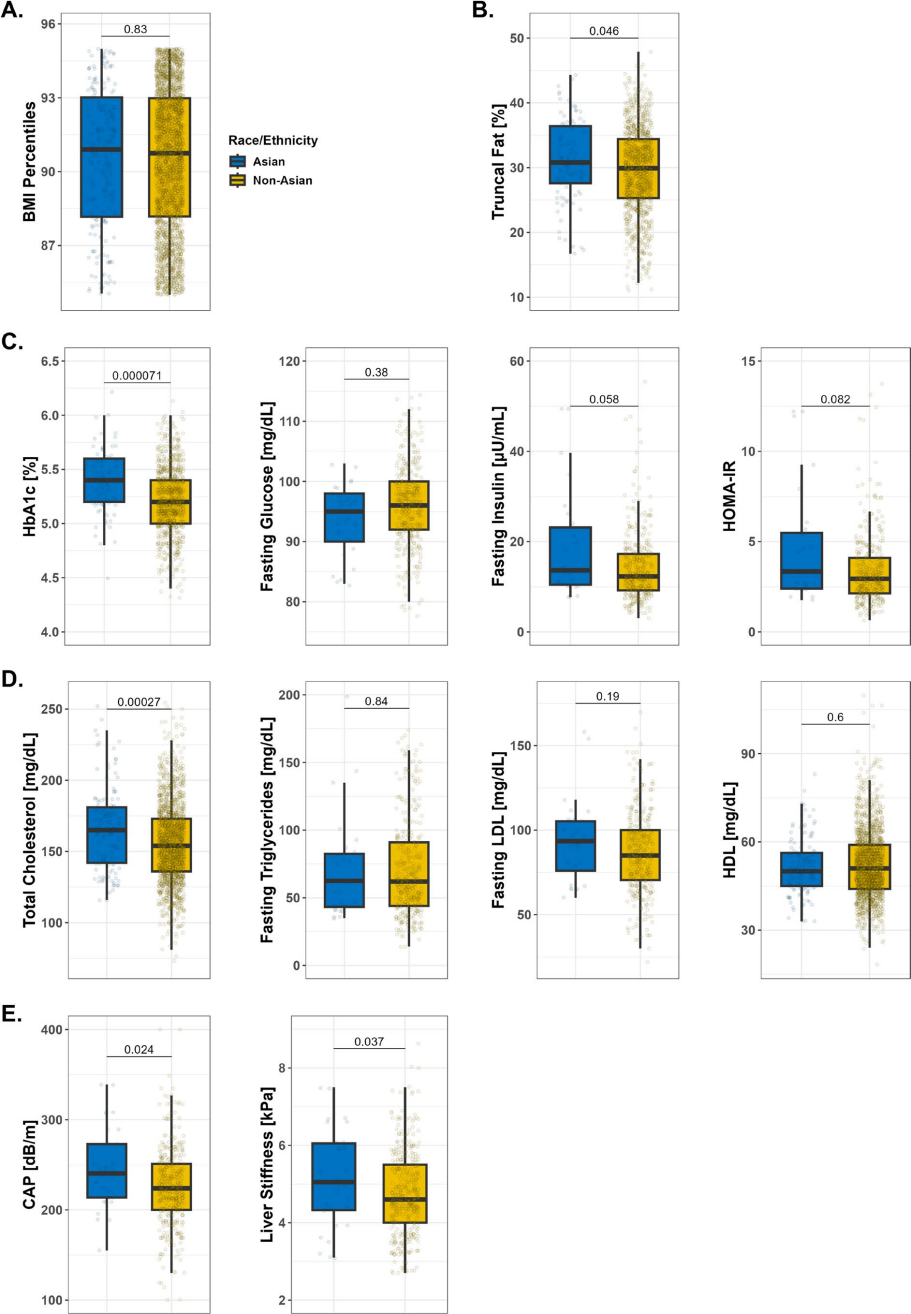
Asian children and adolescents exhibit more adverse metabolic parameters than non‐Asian children and adolescents in the 85–95th BMI percentile range. Comparison of metabolic factors between Asian and non‐Asian children and adolescents in the 85–95th BMI percentile range. (A) BMI percentiles (*n* = 2760). (B) Truncal fat percentage (*n* = 1162). (C) Glucose metabolism and insulin resistance: HbA1c (*n* = 949), values in the 4%–7% range depicted for better visualisation; fasting glucose (*n* = 421), values in the 75–130 mg/dL range depicted; fasting insulin (*n* = 404), values in the 0–60 μU/mL range depicted; HOMA‐IR (*n* = 403), values in the 0–13 range depicted. (D) Lipid profiles: Total cholesterol (*n* = 1781); fasting triglycerides (*n* = 404); fasting LDL‐cholesterol (*n* = 404); HDL‐cholesterol (*n* = 1781). (E) Liver fat and fibrosis: CAP (*n* = 427); liver stiffness (*n* = 427), values in the 3–10 kPa range depicted. Boxplots show median, IQR, and whiskers extending to the largest and smallest values within 1.5 × IQR. Abbreviations: BMI, body mass index; CAP, controlled attenuation parameter; HDL, high‐density lipoprotein; HOMA‐IR, homeostatic model assessment of insulin resistance; IQR, indicates interquartile range; LDL, low‐density lipoprotein.

Further, we compared metabolic factors between Asians and a combined non‐Asian paediatric group in the traditional overweight BMI percentile range (85–95th percentile) (Figure 2). The BMI percentiles were similar between the two groups (median: 90.90th for Asians vs. 90.75th for non‐Asians; *p* = 0.83; Figure 2A). Asians exhibited significantly higher truncal fat percentages (median: 30.8% vs. 29.9%; Figure 2B). HbA1c levels were significantly elevated in Asian participants (median: 5.4% vs. 5.2%; Figure 2C). There was no difference in fasting glucose levels, but a trend toward higher fasting insulin and HOMA‐IR in the Asian group (*p* = 0.058 and *p* = 0.082, respectively; Figure 2C). Regarding lipid profiles, only total cholesterol levels were significantly higher in the Asian group (median: 165 mg/dL vs. 154 mg/dL; Figure 2D). Fasting triglycerides, fasting LDL‐cholesterol and HDL‐cholesterol levels were not significantly different (Figure 2D). The liver disease parameters CAP and liver stiffness were both significantly higher in Asians (median: 240.5 dB/m vs. 224.0 dB/m and 5.2 kPa vs. 4.6 kPa, respectively; Figure 2E). In a supplementary analysis additionally matched for age and sex (Figure S4), overall trends were consistent with the primary results. However, the difference in liver stiffness was no longer statistically significant (*p* = 0.12 vs. *p* = 0.037), although median values remained higher in Asians.

### 3.5 Metabolic Differences Between Asian and Non‐Asian Children and Adolescents in Obesity Range

**FIGURE 3:**
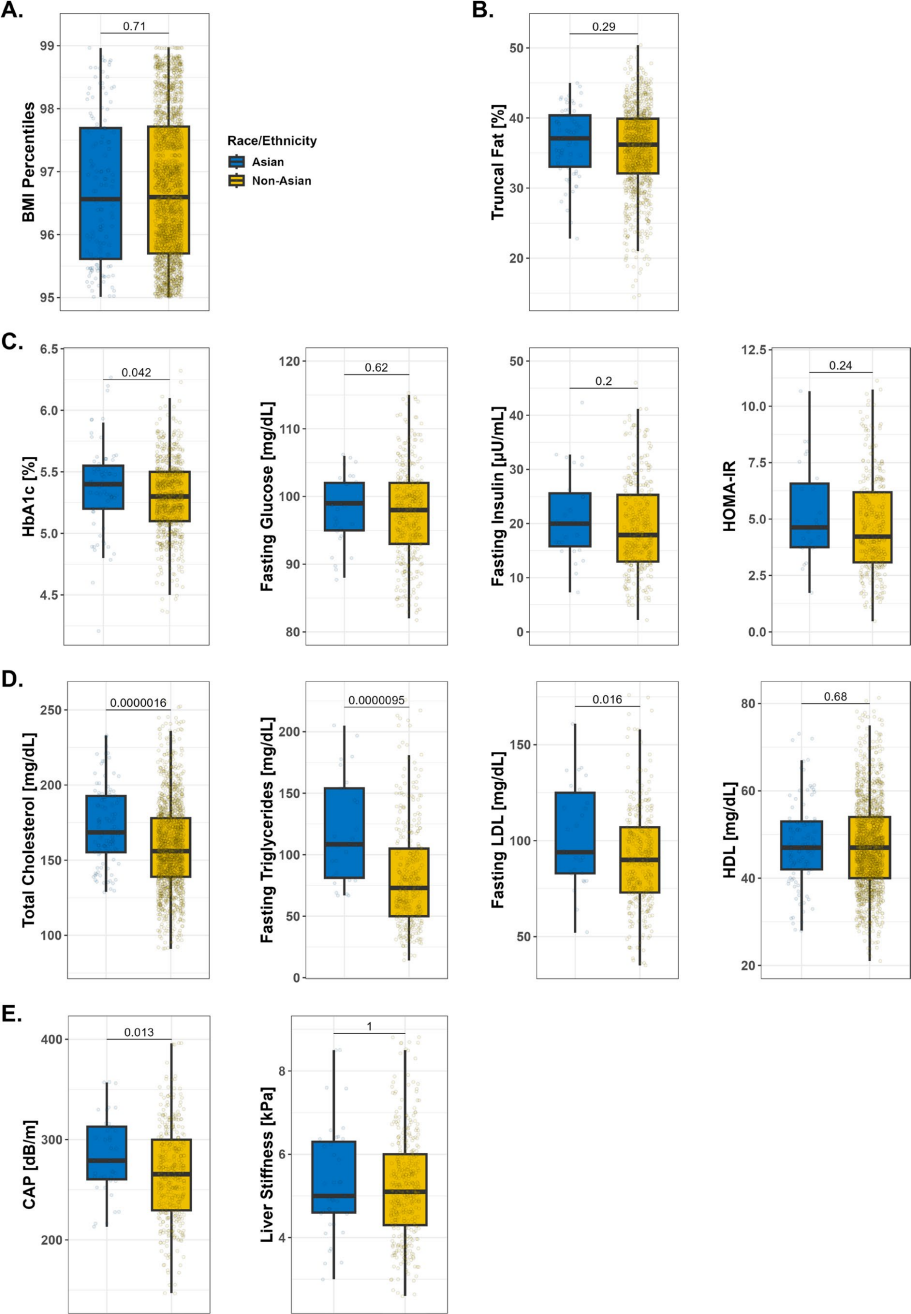
Asian children and adolescents have more severe metabolic dysfunction than non‐Asian children and adolescents in the 95–99th BMI percentile range. Comparison of metabolic factors between Asian and non‐Asian children and adolescents in the 95–99th BMI percentile range after matching for BMI percentiles. (A) BMI percentiles (*n* = 2173). (B) Truncal fat percentage (*n* = 981). (C) Glucose metabolism and insulin resistance: HbA1c (*n* = 804), values in the 4%–6.5% range depicted for better visualisation; fasting glucose (*n* = 371), values in the 80–120 mg/dL range depicted; fasting insulin (*n* = 360), values in the 0–50 μU/mL range depicted; HOMA‐IR (*n* = 360), values in the 0–12 range depicted. (D) Lipid profiles: Total cholesterol (*n* = 1542), values in the 80–260 mg/dL range depicted; fasting triglycerides (*n* = 361), values in the 10–230 mg/dL range depicted; fasting LDL‐cholesterol (*n* = 360); HDL‐cholesterol (*n* = 1542), values in the 20–80 mg/dL range depicted. (E) Liver fat and fibrosis: CAP (*n* = 408), values in the 140–400 dB/m range depicted; liver stiffness (*n* = 408), values in the 2.5–9 kPa range depicted. Boxplots show median, IQR, and whiskers extending to the largest and smallest values within 1.5 × IQR. Abbreviations: BMI, body mass index; CAP, controlled attenuation parameter; HDL, high‐density lipoprotein; HOMA‐IR, homeostatic model assessment of insulin resistance; IQR, indicates interquartile range; LDL, low‐density lipoprotein.

Next, we compared metabolic factors between Asian and non‐Asian children and adolescents in the 95–99th BMI percentile range. However, there was a trend toward lower BMI percentiles in the Asian group compared with non‐Asians (median: 96.56th vs. 96.85th; *p* = 0.10; data not shown). We therefore performed matching for BMI percentiles as above, which allowed for better comparison of metabolic markers between the groups in the 95–99th BMI percentile range (Figure 3). The BMI percentiles showed no difference between the two groups (median: 96.56th for Asians vs. 96.60th for non‐Asians; *p* = 0.71; Figure 3A). Asians did not have higher truncal fat percentages (median: 37.1% vs. 36.2%; *p* = 0.29; Figure 3B). HbA1c levels were significantly elevated in Asian vs. non‐Asian participants (median: 5.4% vs. 5.3%; *p* < 0.001; Figure 3C). There were no statistically significant differences in fasting glucose levels, fasting insulin levels and HOMA‐IR values between the two groups (Figure 3C). With regard to lipid profiles, fasting triglyceride, total cholesterol and fasting LDL‐cholesterol levels were significantly higher in the Asian group (medians: 109 mg/dL vs. 73 mg/dL; 168.5 mg/dL vs. 156.0 mg/dL; and 94 mg/dL vs. 90 mg/dL, respectively), whereas HDL‐cholesterol levels were not significantly different (Figure 3D). The CAP values per Fibroscan were significantly higher in Asians indicating more hepatic steatosis (median: 279 dB/m vs. 263 dB/m; *p* = 0.013), whereas liver stiffness measurements showed no significant difference (Figure 3E). In a supplementary analysis that simultaneously matched for BMI percentile, age and sex (Figure S5), most findings were consistent with the primary results. However, truncal fat became significantly higher in Asians (*p* = 0.035 vs. 0.29 previously), whereas HbA1c lost statistical significance (*p* = 0.082 vs. 0.042). CAP remained significantly higher in Asians (*p* = 0.050).

### 3.6 BMI Percentile Differences Between Asian and Non‐Asian Children and Adolescents After Metabolic Parameter Matching

In order to identify more appropriate cutoffs for overweight and obesity for Asian children and adolescents, we next matched Asian participants within specific BMI percentile ranges (75–85th, 80–90th and 85–95th percentiles) to non‐Asians based on metabolic parameters and compared BMI percentiles between the two groups. Table 2 summarises the matched comparisons within four metabolic subgroups, and Table S2 provides detailed results for all individual comparisons, including median values and differences (HL estimates). Almost all median BMI percentiles were lower in Asian children and adolescents vs. their non‐Asian counterparts after matching for specific metabolic markers (truncal fat, HbA1c, fasting glucose, insulin, HOMA‐IR, total cholesterol, fasting triglycerides, fasting LDL‐cholesterol, HDL‐cholesterol, CAP, or liver stiffness) across the 3 BMI percentile ranges (except liver stiffness in the 80–90th percentile range). In the 75–85th range, the median BMI percentiles were on average 8.73 percentiles lower in Asians compared with non‐Asians (80.01st vs. 89.05th, *p* = 0.002) after averaging all matched BMI percentile subgroup comparisons (for four metabolic marker components: truncal fat; glucose and insulin markers; lipid profiles; and liver imaging) (Table 2). Further, the median BMI percentiles were on average 5.57 percentiles lower in Asians compared with non‐Asians in the 80–90th range (85.26th vs. 91.03rd, *p* = 0.004) after averaging all matched BMI percentile subgroup comparisons. Finally, in the 85–95th range, the median BMI percentiles were on average 3.04 percentiles lower in Asians compared with non‐Asians (90.30th vs. 94.31st, *p* = 0.001) after averaging all matched BMI percentile subgroup comparisons (Table 2). We confirmed that the subset analysis was not biased due to demographic characteristics such as socioeconomic level, as indicated by similar socioeconomic levels with similar poverty rates between the analytic subsets and the full Asian NHANES cohort (not shown).

**TABLE 2:** Corresponding BMI percentiles in non‐Asian children and adolescents matched to Asian children and adolescents by metabolic parameter groups at specific BMI percentile ranges.

Category	*n* ^a^	Asian		Non‐Asian		HL estimate with 95% CI†	*p* ^‡^
BMI percentile range in Asian		BMI percentiles (median)	IQR	BMI percentiles (median)	IQR		
Truncal fat							
75–85	72	80.16	5.16	86.91	16.94	7.86 (4.06–10.51)	< 0.001^§^
80–90	73	84.80	5.08	91.74	14.22	6.28 (3.38–8.32)	< 0.001^§^
85–95	93	90.97	4.94	94.60	9.32	2.94 (1.56–4.20)	< 0.001^§^
Glucose metabolism and insulin resistance							
75–85	135	79.77	4.10	93.02	20.35	11.54 (3.66–14.75)	< 0.001^§^
80–90	147	85.37	5.09	92.00	13.22	5.75 (1.62–8.51)	< 0.001^§^
85–95	163	89.91	4.94	94.63	8.18	3.43 (0.87–5.64)	< 0.001^§^
Lipid profiles							
75–85	237	80.09	4.53	88.35	20.27	8.17 (2.7–13.58)	< 0.001^§^
80–90	253	85.24	5.03	91.32	14.85	5.84 (1.38–8.87)	< 0.001^§^
85–95	319	90.62	4.86	94.13	9.37	2.85 (0.82–4.56)	< 0.001^§^
Liver fat and fibrosis							
75–85	86	80.53	4.11	84.99	16.09	5.54 (1.05–11.54)	0.002^§^
80–90	64	85.23	5.17	88.90	14.19	4.39 (0.43–8.31)	0.005^§^
85–95	62	90.13	5.85	94.31	10.13	2.9 (−0.76–5.46)	< 0.001^§^
Average of 4 metabolic marker components							
75–85		**80.01**	**4.33**	**89.05**	**19.08**	**8.73 (2.68–13.27)**	**0.002** ^§^
80–90		**85.26**	**5.11**	**91.03**	**14.14**	**5.57 (1.4–8.63)**	**0.004** ^§^
85–95		**90.30**	**5.04**	**94.31**	**8.85**	**3.04 (0.61–5.08)**	**0.002** ^§^

*Note:* Data are expressed as median [IQR], with IQR defined as the difference between the 75th percentile (Q3) and the 25th percentile (Q1) (Q3–Q1). Glucose metabolism and insulin resistance include HbA1c, fasting glucose, fasting insulin, and HOMA‐IR; lipid profiles include total cholesterol, fasting triglycerides, fasting LDL‐cholesterol, and HDL‐cholesterol; liver fat and fibrosis include CAP and liver stiffness. Bold indicates the most important information.

Abbreviations: BMI, body mass index; CAP, controlled attenuation parameter; HDL, high‐density lipoprotein; HL, Hodges–Lehmann; HOMA‐IR, homeostatic model assessment of insulin resistance; IQR, indicates interquartile range; LDL, low‐density lipoprotein.

^a^ Number of matched individuals.

^†^ Hodges–Lehmann estimate (Asian–non‐Asian) representing the median difference in BMI percentiles between groups, with corresponding 95% confidence intervals.

^‡^ Calculated using the Wilcoxon rank‐sum test for individual variables and Fisher's method for combining *p* values across metabolic markers.

^§^ Indicates statistically significant differences (*p* < 0.05).

### 3.7 Diagnostic Performance of BMI Percentile Cutoffs (80–95th) for Detecting Metabolic Dysfunction Among Asian Children and Adolescents

In order to find out whether lower BMI percentile cutoffs improve screening for metabolic abnormalities, we performed supplementary analyses restricted to Asian children and adolescents (Table S3). For detecting prediabetes/diabetes, dyslipidaemia and hepatic steatosis, lower BMI percentile thresholds yielded higher sensitivity and good specificity. Of note, the 80th percentile cutoff outperformed the traditional 85th percentile cutoff for detecting metabolic abnormalities within the overweight range, and the 90th percentile cutoff performed better than the 95th percentile cutoff within the obesity range in Asian children and adolescents, as indicated by higher Youden indices and AUCs (Table S3).

## 4 Discussion

Here, we show that compared with other races and ethnicities, Asian children and adolescents exhibit more pronounced metabolic abnormalities, including higher truncal fat percentage, HbA1c, fasting insulin, HOMA‐IR, less favourable lipid profiles and greater hepatic steatosis and stiffness across various BMI percentile ranges. After matching for metabolic markers, the Asian cohort exhibited similar severity of metabolic dysfunction at significantly lower BMI percentiles compared with non‐Asians. Specifically, median BMI percentiles were lower by 8.73 in the 75–85th range, 5.57 in the 80–90th range and 3.04 in the 85–95th range. These findings suggest that current BMI percentile cutoffs may underestimate metabolic health risks in Asian children and adolescents.

We found that Asian children and adolescents have similar truncal fat percentages at significantly lower BMI percentiles compared with non‐Asians (Table 2). This aligns with findings in adults, where Asian populations tend to have higher visceral body fat percentages at lower BMI levels and a greater prevalence of abdominal obesity [12, 27, 28, 29]. Similar patterns have also been reported in Asian paediatric populations residing in Asian countries. For example, studies from East and Southeast Asia have shown that children with lower BMI values already demonstrate higher body fat percentages and adverse metabolic profiles, supporting the need for race and ethnicity‐specific BMI thresholds [30, 31]. Despite dietary and environmental differences between the United States and Asian countries, the consistent finding of elevated metabolic risks at lower BMI levels across settings reinforces the rationale for more sensitive BMI percentile cutoffs in Asian youth. Notably, visceral fat accumulation is particularly harmful, as it is strongly linked to metabolic complications [32]. Moreover, Asian children and adolescents exhibit more pronounced glycemic abnormalities, including elevated HbA1c levels, compared with non‐Asians (Figures 2 and 3; Figure S2), suggesting a greater risk of T2DM. This observation parallels adult studies showing that Asian populations are more susceptible to glucose intolerance and T2DM at lower BMI levels [33] and have a higher prevalence of T2DM even at lower BMI thresholds [34]. In addition, we observed that Asian children and adolescents show higher levels of total cholesterol, fasting triglycerides and fasting LDL‐cholesterol compared with non‐Asians (Figure 3 and S2). This is consistent with adult research demonstrating that South Asians have significantly higher levels of total cholesterol, LDL‐cholesterol and triglycerides, independent of BMI or waist circumference [35]. Furthermore, Asian populations exhibit more adverse lipid profiles compared with White populations [36]. Finally, Asian children and adolescents demonstrated greater hepatic steatosis and liver stiffness, consistent with markers of MASLD, compared with non‐Asians (Figures 2 and 3). This is in line with adult studies that indicate that Asian and South Asian patients with MASLD tend to have an overall lower BMI than other races and ethnicities [37, 38], but exhibit more severe histological findings [38]. Supporting our study, a cohort study of adolescents aged 14–19 years in New York found that Asian adolescents had significantly lower BMI than their non‐Asian counterparts but exhibited higher waist‐to‐height ratios, insulin resistance and triglyceride levels [39]. Moreover, our analyses show that lower BMI percentile thresholds would allow for better screening for metabolic comorbidities, as the 80th percentile cutoff outperformed the traditional 85th percentile cutoff for detecting metabolic abnormalities within the overweight range and the 90th percentile cutoff performed better than the 95th percentile cutoff within the obesity range in Asian children and adolescents (Table S3).

Our study has several limitations. First, the cross‐sectional design of NHANES limits the ability to establish causality or assess temporal relationships between BMI percentiles and metabolic dysfunction. Second, the use of nearest‐neighbour matching based on metabolic parameters resulted in a lower sample size after matching, which may reduce the statistical power, although most of the matched analyses showed statistically significant and also clinically meaningful differences between Asians and non‐Asians. Third, the NHANES dataset does not differentiate among Asian subgroups, preventing more detailed evaluation of Asian subpopulations. However, the widespread adoption of lower BMI cutoffs in adults (mainly BMI ≥ 23.0 kg/m^2^ for overweight, BMI ≥ 25.0 kg/m^2^ for obesity) across Eastern, Southern and Southeastern Asian countries [14, 40] suggests a shared susceptibility to metabolic risks at lower BMI thresholds, despite variations in Asian subpopulations. Larger‐scale, longitudinal studies are warranted to confirm the clinical implications of our findings.

In conclusion, Asian children and adolescents experience greater metabolic risks at a given BMI percentile compared with non‐Asian participants. These findings suggest that current BMI percentile cutoffs may underestimate metabolic health risks in Asian children and adolescents. In our matched analyses, Asians showed comparable levels of metabolic dysfunction at BMI percentiles that were approximately 3–9 points lower than those of non‐Asians, across various BMI percentile ranges. Based on these consistent percentile differences, lowering the thresholds for overweight to approximately the 80th percentile and for obesity to approximately the 90th percentile may better capture early metabolic risks and improve screening in the Asian paediatric population.

## Author Contributions

S.‐J.H. directly accessed and verified the underlying data reported in the manuscript, analyzed, and interpreted the data, and wrote the manuscript. X.Z. directly accessed and verified the underlying data reported in the manuscript, and edited the manuscript. P.H. designed the study, developed the method, supervised the work, directly accessed and verified the underlying data reported in the manuscript, analyzed, and interpreted the data, and edited the manuscript. All authors have approved the submitted version.

## Funding

This work was supported by National Institutes of Health (NIH) grant K12 HD105271, University of California San Diego Altman Clinical and Translational Research Institute (ACTRI)/NIH grant KL2TR001444, and Pinnacle Research Award in Liver Diseases Grant #PNC22‐159963 from the American Association for the Study of Liver Diseases Foundation (to PH), and services provided by NIH center P30 DK120515. The funding sources had no role in the design and conduct of the study; collection, management, analysis, and interpretation of the data; preparation, review, or approval of the manuscript; or decision to submit the manuscript for publication.

## Ethics Statement

The authors have nothing to report.

## Conflicts of Interest

P.H.'s institution, the University of California San Diego, has received research support from Nterica Bio. S.‐J.H. and X.Z. authors declare no conflicts of interest.

## Supporting information


**Table S1:** Age and sex distributions of participants by race and ethnicity.
**Table S2:** Corresponding BMI percentiles in non‐Asian children and adolescents matched to Asian children and adolescents based on metabolic parameters at specific BMI percentile ranges.
**Table S3:** Diagnostic performance of BMI percentile cutoffs (80th‐95th) for detecting prediabetes/diabetes, dyslipidaemia, and hepatic steatosis among Asian children and adolescents.
**Figure S1:** Asian children and adolescents have a significantly lower BMI percentile than all other races/ethnicities and might still show worse metabolic markers than other races/ethnicities.
**Figure S2:** Asian children and adolescents demonstrate worse metabolic parameters than non‐Asian children and adolescents.
**Figure S3:** Asian children and adolescents demonstrate worse metabolic parameters than non‐Asian children and adolescents.
**Figure S4:** Asian children and adolescents exhibit worse metabolic parameters than non‐Asian children and adolescents in the 85–95th BMI percentile range.
**Figure S5:** Asian children and adolescents have more severe metabolic dysfunction than non‐Asian children and adolescents in the 95–99th BMI percentile range.

## Data Availability

The data used in this study are publicly available from the Centers for Disease Control and Prevention (CDC) National Health and Nutrition Examination Survey (NHANES) database: https://www.cdc.gov/nchs/nhanes.
