# Supplementary material for: BMI Percentile Cutoffs for Overweight and Obesity Are Set Too High in Terms of Adiposity and Metabolic Markers for Asian Children and Adolescents
Source: Pediatr Obes. 2025 Dec 10;21(1):e70075. doi: 10.1111/ijpo.70075 (PMC12690340; doi:10.1111/ijpo.70075)
Supplement: Supplementary file 1 — Table S1: Age and sex distributions of participants by race and ethnicity. Table S2: Corresponding BMI percentiles in non‐Asian children and adolescents matched to Asian children and adolescents based on metabolic parameters at specific BMI percentile ranges. Table S3: Diagnostic performance of BMI percentile cutoffs (80th‐95th) for detecting prediabetes/diabetes, dyslipidaemia, and hepatic steatosis among Asian children and adolescents. Figure S1: Asian children and adolescents have a significantly lower BMI percentile than all other races/ethnicities and might still show worse metabolic markers than other races/ethnicities. Figure S2: Asian children and adolescents demonstrate worse metabolic parameters than non‐Asian children and adolescents. Figure S3: Asian children and adolescents demonstrate worse metabolic parameters than non‐Asian children and adolescents. Figure S4: Asian children and adolescents exhibit worse metabolic parameters than non‐Asian children and adolescents in the 85–95th BMI percentile range. Figure S5: Asian children and adolescents have more severe metabolic dysfunction than non‐Asian children and adolescents in the 95–99th BMI percentile range. [file IJPO-21-e70075-s001.pdf]

BMI percentile cutoffs for overweight and obesity are set too high in terms of adiposity and metabolic markers for Asian children and adolescents

Suk-Jin Hong, M.D., Ph.D., Xinlian Zhang, Ph.D., Phillipp Hartmann, M.D., M.A.S.

Correspondence:

Phillipp Hartmann, M.D., M.A.S., D.A.B.O.M., Department of Pediatrics, Division of Pediatric Gastroenterology, Hepatology, and Nutrition, University of California San Diego, 9500 Gilman Dr, La Jolla, CA 92093-0984, Tel: 858-966-4003, Fax: 858-560-6798, Email: phhartmann@health.ucsd.edu

Table of Contents

Supplementary Table **2**

Table S1 .... **2**

Table S2 .... **3**

Table S3 .... **5**

Supplementary Figures **6**

Figure S1 .... **6**

Figure S2 .... **8**

Figure S3 .... **10**

Figure S4 .... **12**

Figure S5 .... **14**

Supplementary Table

Table S1. Age and Sex Distributions of Participants by Race and Ethnicity.

Race and Ethnicity	Age [years]	Sex, n	
		Male	Female
Non-Hispanic Asian	10.5 [6.5;15.5]	853 (50.6%)	832 (49.4%)
Non-Hispanic White	9.5 [5.5;14.5]	2,432 (51.4%)	2,298 (48.6%)
Non-Hispanic Black	9.5 [5.5;13.5]	2,283 (51.7%)	2,132 (48.3%)
Mexican American	9.5 [6.5;14.5]	1,500 (46.9%)	1,699 (53.1%)
Other Hispanic	9.5 [5.5;13.5]	919 (52.8%)	822 (47.2%)
Other Race	9.5 [5.5;13.5]	675 (50.9%)	651 (49.1%)

Values are presented as median and in brackets the first and third quartiles.

Table S2. Corresponding BMI Percentiles in non-Asian Children and Adolescents Matched to Asian Children and Adolescents Based on Metabolic Parameters at Specific BMI Percentile Ranges.

Category		Asian		Non-Asian			
BMI percentile range in Asian	n*	BMI percentiles (Median)	IQR	BMI percentiles (Median)	IQR	HL estimate with 95% CI†	P-value‡
**Truncal Fat**							
**75-85**	72	80.16	5.16	86.91	16.94	7.86 (4.06-10.51)	<0.001§
**80-90**	73	84.80	5.08	91.74	14.22	6.28 (3.38-8.32)	<0.001§
**85-95**	93	90.97	4.94	94.60	9.32	2.94 (1.56-4.2)	<0.001§
**HbA1c**							
**75-85**	66	80.82	4.62	93.55	19.57	12.6 (10-14.45)	<0.001§
**80-90**	72	85.12	5.03	95.01	11.57	8.33 (6.08-9.95)	<0.001§
**85-95**	77	90.40	4.86	95.19	7.05	3.94 (2.5-5.29)	<0.001§
**Fasting Glucose**							
**75-85**	23	79.25	3.84	94.31	19.78	11.75 (-0.3-15.91)	0.059
**80-90**	27	85.46	4.84	92.94	14.60	5.91 (0.74-8.54)	0.032§
**85-95**	30	89.52	5.41	93.79	7.18	3.43 (0.97-5.73)	0.008§
**Fasting Insulin**							
**75-85**	23	79.25	3.84	91.20	21.70	10.28 (1.27-13.89)	0.033§
**80-90**	24	85.54	5.40	88.05	13.51	3.02 (-1.98-7.04)	0.2
**85-95**	28	89.82	4.56	94.91	10.31	2.92 (-0.86-5.91)	0.12
**HOMA-IR**							
**75-85**	23	79.25	3.84	90.22	18.64	9.74 (1.6-13.81)	0.028§
**80-90**	24	85.54	5.40	90.48	13.86	5.53 (0.83-8.99)	0.031§
**85-95**	28	89.82	4.56	93.82	5.69	2.99 (0.79-5.53)	0.012§
**Total Cholesterol**							
**75-85**	97	80.85	5.21	94.27	15.60	13 (10.62-14.67)	<0.001§
**80-90**	103	84.69	4.45	92.05	13.62	6.5 (4.3-8.35)	<0.001§
**85-95**	131	91.43	5.10	93.12	10.00	1.66 (0.3-2.92)	0.02§
**Fasting Triglycerides**							
**75-85**	21	79.25	3.71	89.02	20.10	9.58 (1.92-16.18)	0.015§
**80-90**	23	85.76	5.55	91.09	15.64	4.81 (-1.82-9.35)	0.13
**85-95**	28	89.82	4.56	95.35	6.88	4.34 (1.93-6.44)	<0.001§
**Fasting LDL**							
**75-85**	20	79.41	3.77	81.91	26.58	2.38 (-6.03-12.9)	0.58
**80-90**	23	85.76	5.55	92.12	14.34	6.93 (0.44-10.38)	0.031§
**85-95**	28	89.82	4.56	94.65	9.60	3.39 (0.53-5.58)	0.031§

**HDL**							
**75-85**	99	80.85	5.40	88.18	18.80	7.71 (4.28-10.59)	<0.001[§]
**80-90**	104	84.75	4.58	90.00	15.81	5.12 (2.59-7.4)	<0.001[§]
**85-95**	132	91.40	5.22	93.40	11.00	2.03 (0.52-3.3)	0.014[§]
**CAP**							
**75-85**	30	80.85	4.40	85.61	13.22	5.79 (1.93-10.62)	0.006[§]
**80-90**	33	85.46	5.17	93.28	13.23	7.72 (3.51-9.91)	0.001[§]
**85-95**	32	89.52	5.99	95.46	6.51	3.91 (1.92-6.6)	<0.001[§]
**Liver Stiffness**							
**75-85**	56	80.21	3.82	84.36	18.95	5.3 (0.18-12.47)	0.043[§]
**80-90**	31	84.99	5.17	84.52	15.15	1.06 (-2.66-6.72)	0.58
**85-95**	30	90.74	5.71	93.16	13.75	1.88 (-3.43-4.32)	0.47
**Average of all Metabolic Markers**							
**75-85**		**80.01**	**4.33**	**89.05**	**19.08**	**8.73 (2.68-13.27)**	**0.002**[§]
**80-90**		**85.26**	**5.11**	**91.03**	**14.14**	**5.57 (1.4-8.63)**	**0.004**[§]
**85-95**		**90.30**	**5.04**	**94.31**	**8.85**	**3.04 (0.61-5.08)**	**0.002**[§]

Data are expressed as median [IQR], with IQR defined as the difference between the 75th percentile (Q3) and the 25th percentile (Q1) (Q3–Q1).

BMI, Body Mass Index; CAP, Controlled Attenuation Parameter; CI, Confidence Interval; HDL, High-Density Lipoprotein; HL, Hodges–Lehmann, HOMA-IR, Homeostatic Model Assessment of Insulin Resistance; IQR indicates interquartile range; LDL, Low-Density Lipoprotein.

* Number of matched individuals.

† Hodges–Lehmann estimate (Asian – non-Asian) representing the median difference in BMI percentiles between groups, with corresponding 95% confidence intervals.

‡ Calculated using the Wilcoxon rank-sum test for individual variables and Fisher's method for combining p-values across metabolic markers.

§ Indicates statistically significant differences ($p < 0.05$).

Supplementary Table S3. Diagnostic Performance of BMI Percentile Cutoffs (80th–95th) for Detecting Prediabetes/Diabetes, Dyslipidemia, and Hepatic Steatosis Among Asian Children and Adolescents.

Outcome	BMI Percentile Threshold	Sensitivity	Specificity	Youden Index	AUC (95% CI)
Prediabetes or Diabetes (n=628)	80	0.60	0.72	0.32	0.660 (0.588-0.732)
	85	0.52	0.77	0.29	0.646 (0.572-0.719)
	90	0.38	0.82	0.20	0.598 (0.527-0.669)
	95	0.23	0.88	0.11	0.554 (0.493-0.616)
Dyslipidemia (n=1,026)	80	0.39	0.77	0.16	0.579 (0.550-0.607)
	85	0.33	0.81	0.14	0.571 (0.544-0.598)
	90	0.27	0.86	0.13	0.565 (0.539-0.590)
	95	0.18	0.93	0.10	0.551 (0.530-0.571)
Hepatic Steatosis (n=280)	80	0.89	0.76	0.66	0.830 (0.773-0.886)
	85	0.84	0.82	0.66	0.832 (0.769-0.896)
	90	0.79	0.88	0.67	0.837 (0.768-0.906)
	95	0.66	0.93	0.58	0.792 (0.714-0.870)

Prediabetes and diabetes were defined by HbA1c ≥ 5.7%; dyslipidemia by total cholesterol ≥ 170 mg/dL or HDL < 40 mg/dL; and hepatic steatosis by controlled attenuation parameter ≥ 277 dB/m. AUC, area under the receiver operating characteristic curve; CI, confidence interval.

Supplementary Figures

Figure S1

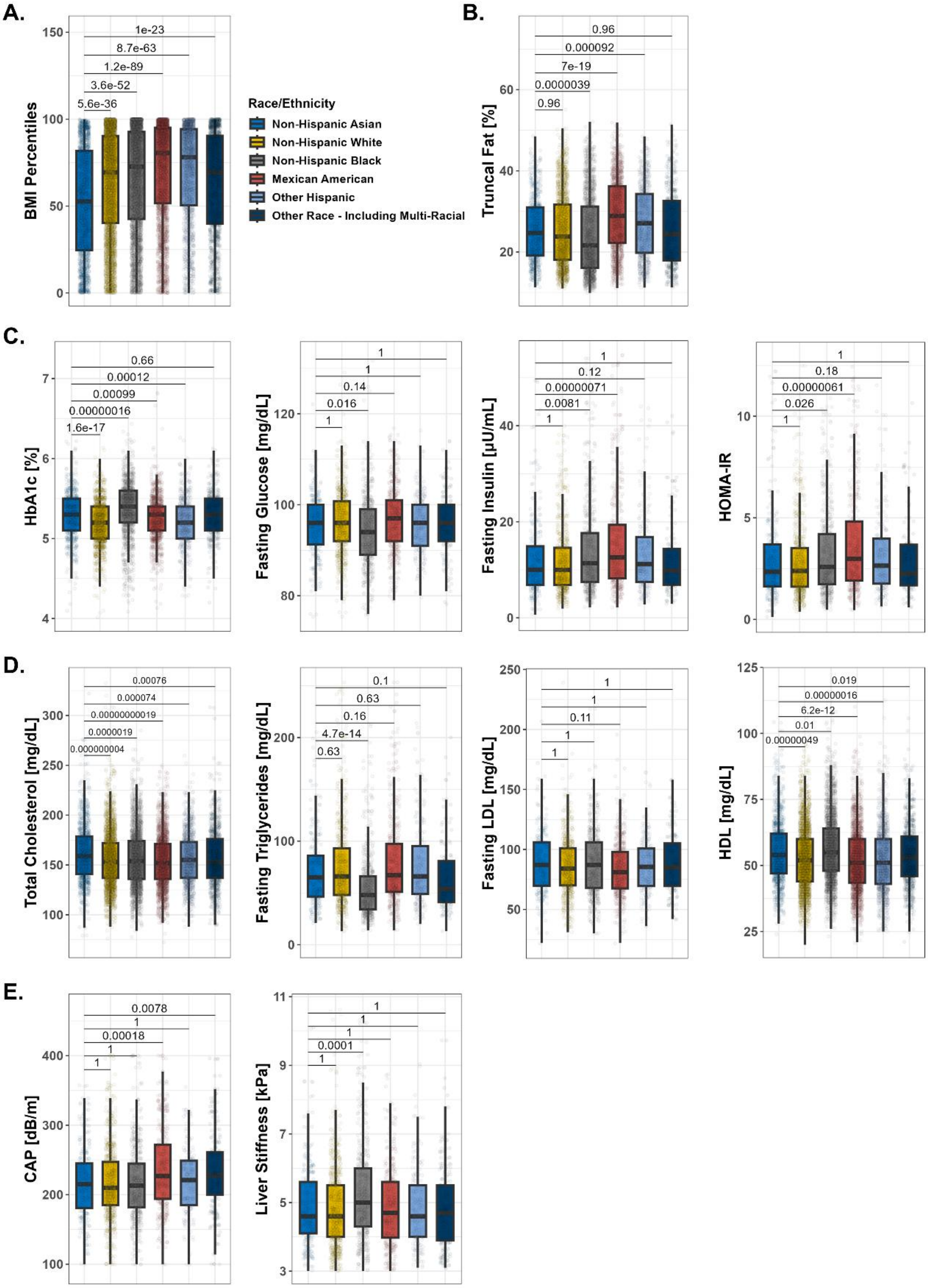

Figure S1. Asian children and adolescents have a significantly lower BMI percentile than all other races/ethnicities and might still show worse metabolic markers than other races/ethnicities. Comparison of metabolic factors by ethnic

groups in children and adolescents including all BMI percentiles. (A) BMI percentiles (n=17,096). (B) Truncal fat percentage (n=6,761). (C) Glucose metabolism and insulin resistance: HbA1c (n=5,421), values in 4–7.5% range depicted for better visualization; fasting glucose (n=2,521), values in 75–135 mg/dL range depicted; fasting insulin (n=2,413), values in 0–55 μU/mL range depicted; HOMA-IR (n=2,412), values in 0–13 range depicted. (D) Lipid profiles: Total cholesterol (n=10,388), values in 70–330 mg/dL range depicted; fasting triglycerides (n=2,428), values in 0–250 mg/dL range depicted; fasting LDL-cholesterol (n=2,423); HDL-cholesterol (n=10,389), values in 20–120 mg/dL range depicted. (E) Liver fat and fibrosis: CAP (n=2,520), values in 100–400 dB/m range depicted; liver stiffness (n=2,522), values in 3–11 kPa range depicted. Points represent individual participants; boxes represent IQR; central lines correspond to the medians; whiskers extend to the largest and smallest values within 1.5 × IQR. P-values were adjusted for multiple comparisons using the Holm method, with non-Hispanic Asians serving as the reference group. Statistical significance is indicated by $p<0.05$. BMI, Body Mass Index; CAP, Controlled Attenuation Parameter; HDL, High-Density Lipoprotein; HOMA-IR, Homeostatic Model Assessment of Insulin Resistance; IQR indicates interquartile range; LDL, Low-Density Lipoprotein.

Figure S2

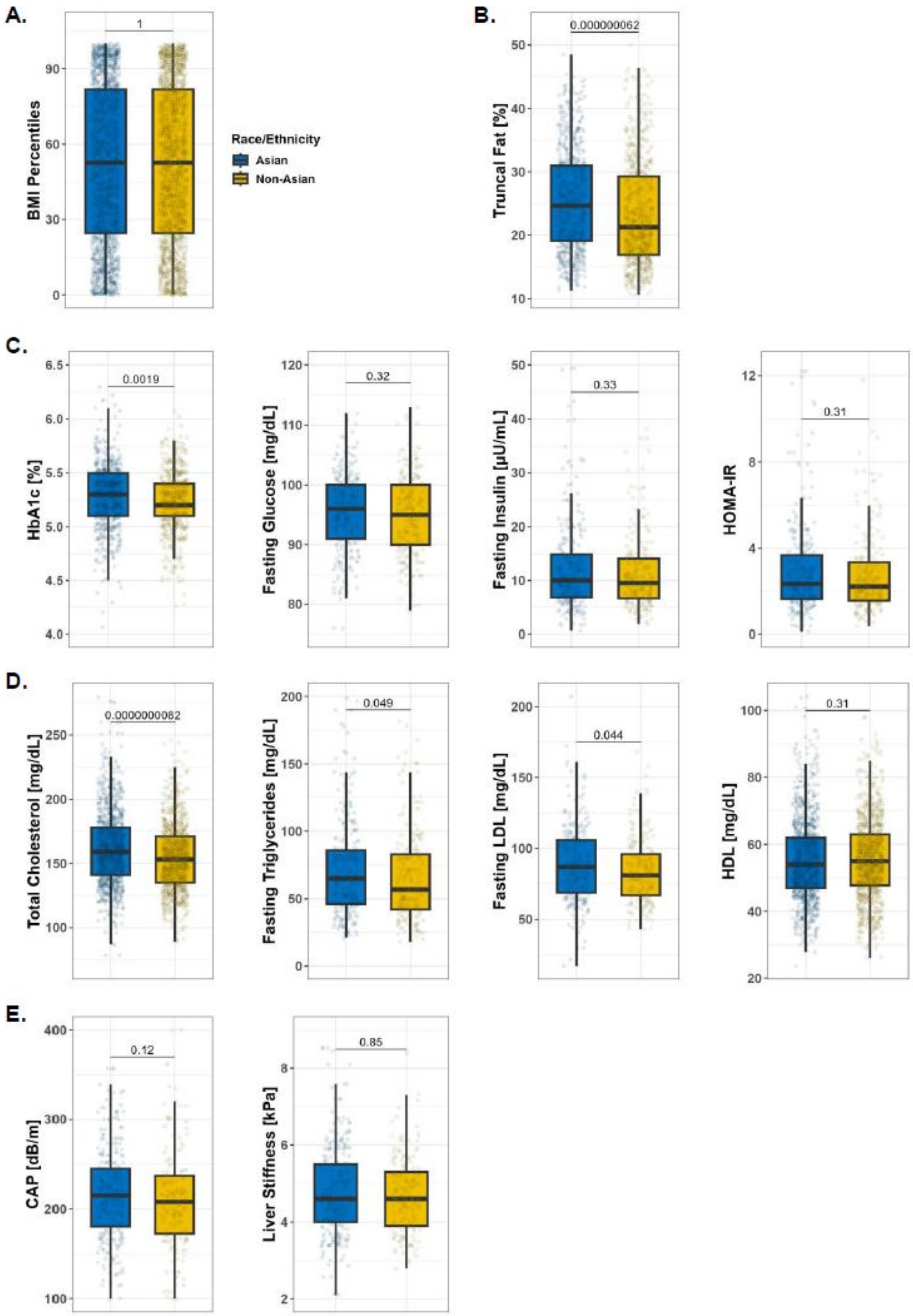

Figure S2. Asian children and adolescents demonstrate worse metabolic parameters than non-Asian children and adolescents. Comparison of metabolic

factors between Asian and non-Asian children and adolescents including all BMI percentiles after matching for BMI percentiles. (A) BMI percentiles (n=3,370). (B) Truncal fat percentage (n=1,367). (C) Glucose metabolism and insulin resistance: HbA1c (n=1,131), values in 4–6.5% range depicted for better visualization; fasting glucose (n=502), values in 70–120 mg/dL range depicted; fasting insulin (n=480), values in 0–50 μU/mL range depicted; HOMA-IR (n=479), values in 0–12.5 range depicted. (D) Lipid profiles: Total cholesterol (n=1,990), values in 70–280 mg/dL range depicted; fasting triglycerides (n=481), values in 0–200 mg/dL range depicted; fasting LDL-cholesterol (n=480); HDL-cholesterol (n=1,990). (E) Liver fat and fibrosis: CAP (n=455); liver stiffness (n=455), values in 2–9 kPa range depicted. Points represent individual participants; boxes represent IQR; central lines correspond to the medians; whiskers extend to the largest and smallest values within 1.5 × IQR. Statistical significance is indicated by $p<0.05$.

BMI, Body Mass Index; CAP, Controlled Attenuation Parameter; HDL, High-Density Lipoprotein; HOMA-IR, Homeostatic Model Assessment of Insulin Resistance; IQR indicates interquartile range; LDL, Low-Density Lipoprotein.

Figure S3

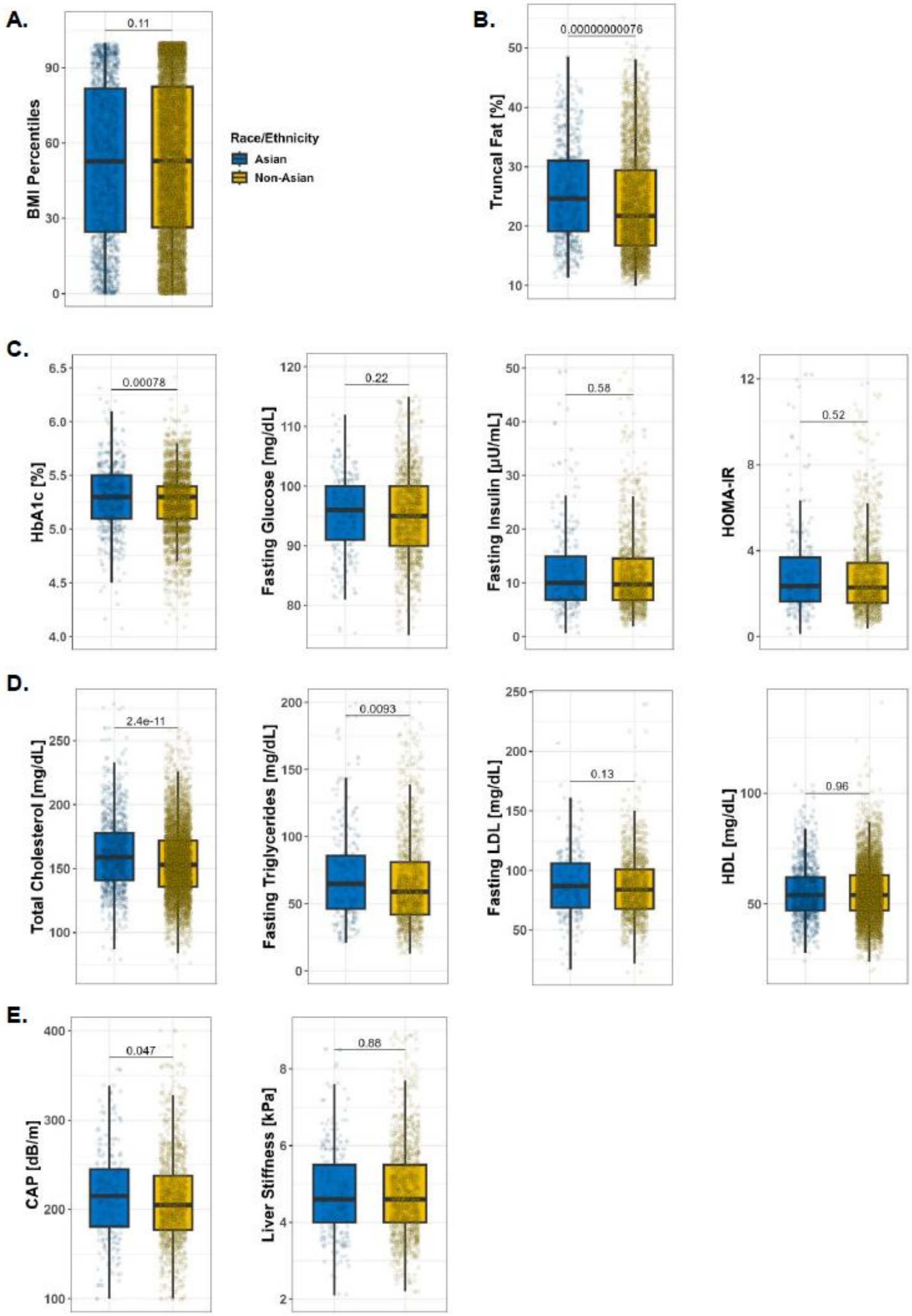

Figure S3. Asian children and adolescents demonstrate worse metabolic parameters than non-Asian children and adolescents. Comparison of metabolic

factors between Asian and non-Asian children and adolescents including all BMI percentiles after matching for BMI percentile, age, and sex. (A) BMI percentiles (n = 19,848). (B) Truncal fat percentage (n = 4,206). (C) Glucose metabolism and insulin resistance: HbA1c (n = 3,502, values in 4–6.5 % range depicted for better visualization); fasting glucose (n = 1,631, values in 75–120 mg/dL range depicted); fasting insulin (n = 1,552, values in 0–50 µU/mL range depicted); HOMA-IR (n = 1,552, values in 0–12.5 range depicted). (D) Lipid profiles: total cholesterol (n = 6,172, values in 70–280 mg/dL range depicted); fasting triglycerides (n = 1,565, values in 0–200 mg/dL range depicted); fasting LDL-cholesterol (n = 1,561); HDL-cholesterol (n = 6,172). (E) Liver fat and fibrosis: CAP (n = 1,566) and liver stiffness (n = 1,568, values in 2–9 kPa range depicted). Points represent individual participants; boxes represent IQR; central lines correspond to the medians; whiskers extend to the largest and smallest values within 1.5 × IQR. Statistical significance is indicated by $p<0.05$.

BMI, Body Mass Index; CAP, Controlled Attenuation Parameter; HDL, High-Density Lipoprotein; HOMA-IR, Homeostatic Model Assessment of Insulin Resistance; IQR indicates interquartile range; LDL, Low-Density Lipoprotein.

Figure S4

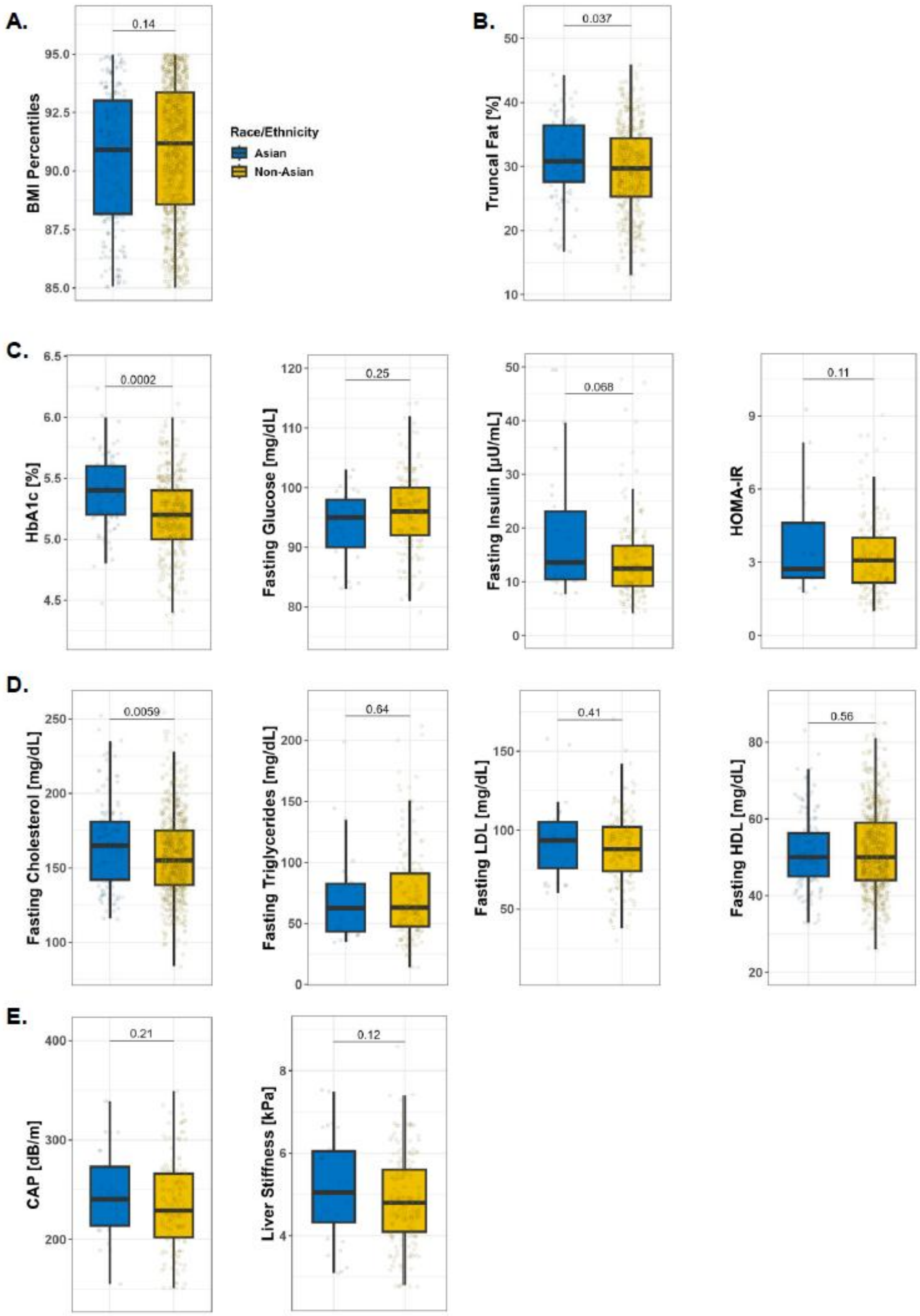

Figure S4. Asian children and adolescents exhibit worse metabolic parameters than non-Asian children and adolescents in the 85th-95th BMI percentile range. Comparison of metabolic factors between Asian and non-Asian children and

adolescents in the 85th–95th BMI percentile range after matching for BMI percentile, age, and sex. (A) BMI percentiles (n = 9,848). (B) Truncal fat percentage (n = 4,206). (C) Glucose metabolism and insulin resistance: HbA1c (n = 3,502, values in 4.2–6.4 % range depicted for better visualization); fasting glucose (n = 1,631, values in 75–120 mg/dL range depicted); fasting insulin (n = 1,552, values in 0–50 μU/mL range depicted); HOMA-IR (n = 1,552, values in 0–11 range depicted). (D) Lipid profiles: total cholesterol (n = 6,172, values in 80–260 mg/dL range depicted); fasting triglycerides (n = 1,565, values in 10–230 mg/dL range depicted); fasting LDL-cholesterol (n = 1,561, values in 10–180 mg/dL range depicted); HDL-cholesterol (n = 6,172, values in 20–90 mg/dL range depicted). (E) Liver fat and fibrosis: CAP (n = 1,566, values in 140–410 dB/m range depicted); liver stiffness (n = 1,568, values in 2.5–9 kPa range depicted). Boxplots show median, IQR, and whiskers extending to the largest and smallest values within 1.5 × IQR.

BMI, Body Mass Index; CAP, Controlled Attenuation Parameter; HDL, High-Density Lipoprotein; HOMA-IR, Homeostatic Model Assessment of Insulin Resistance; IQR indicates interquartile range; LDL, Low-Density Lipoprotein.

Figure S5

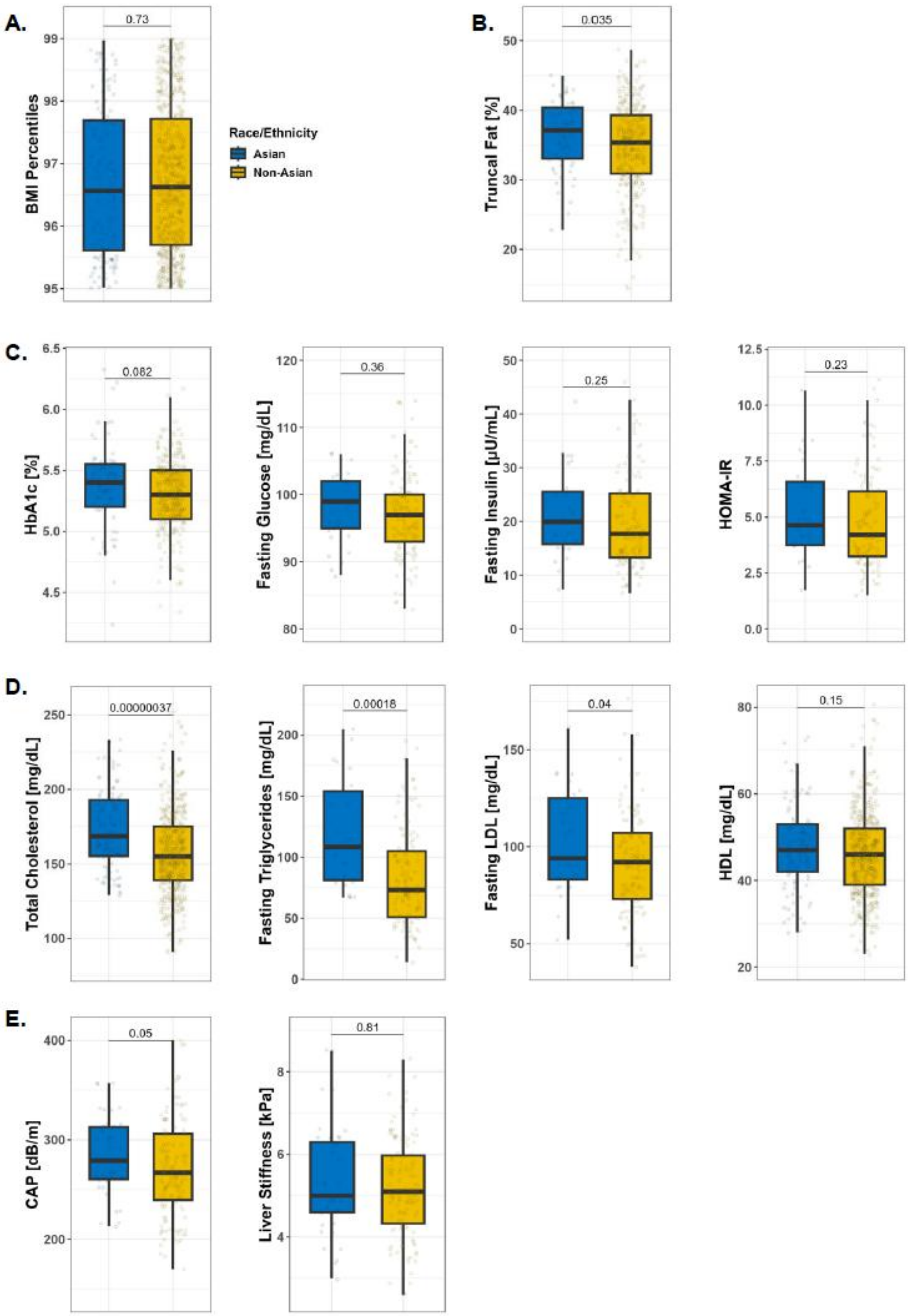

Figure S5. Asian children and adolescents have more severe metabolic dysfunction than non-Asian children and adolescents in the 95th–99th BMI

percentile range. Comparison of metabolic factors between Asian and non-Asian children and adolescents in the 95th–99th BMI percentile range after matching for BMI percentile, age, and sex. (A) BMI percentiles (n = 808). (B) Truncal fat percentage (n = 406). (C) Glucose metabolism and insulin resistance: HbA1c (n = 371, values in 4.2–6.4 % range depicted for better visualization); fasting glucose (n = 160, values in 80–120 mg/dL range depicted); fasting insulin (n = 153, values in 0–50 µU/mL range depicted); HOMA-IR (n = 153, values in 0–12 range depicted). (D) Lipid profiles: total cholesterol (n = 603, values in 80–260 mg/dL range depicted); fasting triglycerides (n = 154, values in 10–230 mg/dL range depicted); fasting LDL-cholesterol (n = 154); HDL-cholesterol (n = 603, values in 20–82 mg/dL range depicted). (E) Liver fat and fibrosis: CAP (n = 180, values in 140–410 dB/m range depicted); liver stiffness (n = 180, values in 2.5–9 kPa range depicted). Boxplots show median, IQR, and whiskers extending to the largest and smallest values within 1.5 × IQR.

BMI, Body Mass Index; CAP, Controlled Attenuation Parameter; HDL, High-Density Lipoprotein; HOMA-IR, Homeostatic Model Assessment of Insulin Resistance; IQR indicates interquartile range; LDL, Low-Density Lipoprotein.
